# The role of sawdust Ca-biochar on the phosphate adsorption: an optimization through a 2^3^ experimental design

**DOI:** 10.1007/s11356-026-37966-8

**Published:** 2026-07-07

**Authors:** Amanda Ribeiro da Rocha, Juliana Schultz, Antonio Salvio Mangrich, Glaucia Pantano

**Affiliations:** UFPR: Universidade Federal Do Parana, Curitiba, Brazil

**Keywords:** Biomass, Phosphorus, Pyrolysis, Eutrophication

## Abstract

Phosphorus (P) is a vital element for sustaining life; however, its excessive discharge into aquatic systems has accelerated eutrophication, while global reserves of phosphate rock approach depletion. Developing efficient materials for phosphate removal and recovery is essential to ensure food and water security. In this study, pine sawdust, an abundant industrial by-product in Brazil, was converted into calcium-modified biochars using both batch (tubular furnace) and continuous-flow pyrolysis. A 2^3^ factorial design was applied to optimize modification parameters (time, concentration, and biomass mass). Phosphate removal efficiencies ranged from 13 to 97%, with the statistical model explained 99% of the response variation. Characterization by SEM/EDS and BET revealed fissures, porosity, and effective Ca incorporation, while FTIR identified persistent oxygen-containing functional groups (–OH, C = O, C–O–C), Raman spectroscopy indicated partially graphitized carbon structures with defect-rich surfaces (I_D_/I_G_ = 2.01–2.62), and XRD confirmed the presence of CaO, Ca(OH)_2_, and CaCO_3_. Equilibrium data fitted Langmuir, Freundlich, and Sips models, with maximum adsorption of 158.4 mg g⁻^1^. The combined characterization and adsorption results indicated that phosphate removal occurred predominantly through calcium-mediated mechanisms, including surface complexation and calcium-phosphate precipitation. Biochars produced in the pilot-scale continuous-flow furnace exhibited adsorption efficiencies comparable to those obtained in the laboratory-scale tubular furnace, demonstrating the feasibility of continuous production while maintaining adsorption performance. The results highlight calcium-modified sawdust biochars as promising materials for phosphate removal and recovery, while the formation of phosphorus-enriched biochars creates opportunities for nutrient recycling within a circular economy framework.

## Introduction

Phosphorus (P) is an essential nutrient for sustaining life on Earth. It is a structural component for biological systems, and it is indispensable agriculture and industry (Cakmak et al. 2022; Krüger and Adam 2017; Zhang et al. 2025). As one of the three macronutrients in NPK fertilizers, phosphorus ensures crop productivity, root development, and food production. Beyond agriculture, it is also employed in detergents, pharmaceuticals, plastics, electronics, and other industrial applications (Jørgensen et al. 2018; Zhang et al. 2024).

Despite its strategic importance, phosphorus is a non-renewable resource obtained mainly from phosphate rock deposits, which are finite and unevenly distributed worldwide. Approximately 84% of exploitable reserves are concentrated in Morocco and neighboring regions, Egypt, China, and the USA, creating concerns regarding supply security and geopolitical dependence (Houssini et al. 2023; USGS, 2025). Consequently, phosphorus has been recognized as a Critical Raw Material (CRM) by the European Commission, highlighting the urgency of developing efficient recovery and recycling strategies (EC, 2023; Glaser and Lehr 2019).

Intensive mining, fertilizer overuse, and the absence of effective recycling strategies have led to decreasing of phosphate rocks and increasing accumulation of P in aquatic systems. When discharged in treated and untreated sewage, or through agricultural runoff, phosphorus reaches rivers, lakes, and reservoirs that drive eutrophication At the same time, inefficient phosphorus management has resulted in increasing phosphate losses to aquatic environments through agricultural runoff, industrial effluents, and treated or untreated wastewater discharges. Excessive phosphorus inputs stimulate eutrophication, one of the most significant environmental challenges affecting freshwater systems worldwide (Di Capua et al. 2022; Le Moal et al. 2019).

The over-enrichment of nutrients promotes excessive algal growth, reduces light penetration, accelerates oxygen depletion during organic matter degradation, and ultimately leads to the formation of hypoxic or anoxic “dead zones,” compromising biodiversity, ecosystem services, and water security (Bacelo et al. 2020; Charlton et al. 2018; Suresh et al. 2023). This paradoxical scenario, in which phosphorus simultaneously represents a critical resource and a major pollutant, highlights the importance of technologies capable of both removing and recovering phosphate from aqueous systems, thereby supporting circular economy principles and enhancing food and water security.

Several technologies have been investigated for phosphate removal and recovery, including chemical precipitation using iron, aluminum, or calcium salts, membrane processes, ion exchange, clay-based materials, engineered mineral amendments, layered double hydroxides (LDHs), metal oxides, activated carbons, and biosorbents (Wilfert et al. 2015; Xavier et al. 2024). While many of these materials exhibit high phosphate removal efficiencies, they often suffer from limitations related to operational costs, regeneration difficulties, secondary waste generation, or limited scalability. In recent years, adsorption-based technologies have gained increasing attention due to their operational simplicity, economic feasibility, and potential for phosphorus recovery and reuse (Nadagouda et al. 2024; Zheng et al. 2023).

Among the adsorbent materials, biochar has emerged as one of the most promising alternatives due to its low production cost, feedstock versatility, environmental sustainability, and tunable physicochemical properties. It is a stable porous carbonaceous material produced through biomass pyrolysis under oxygen-limited conditions, typically between 300 and 900 °C (Amalina et al. 2023; Hassan et al. 2020; Wang et al. 2024). Phosphate sorption occurs through a combination of physisorption and chemisorption mechanisms, including electrostatic attraction, ligand exchange, ion exchange, surface complexation, and precipitation (Sizmur et al. 2017).

Pine sawdust represents a particularly attractive feedstock for biochar production in Brazil due to its abundance and continuous availability. Paraná State alone accounts for approximately 42% of the national pine plantation area, and large quantities of sawdust are generated as a by-product of the forestry and pulp industries (IBÁ, 2024). The conversion of this residue into value-added biochar contributes simultaneously to waste valorization, resource efficiency, and sustainable material development. However, the performance of biochar is strongly influenced by biomass characteristics, metal modification strategies, pyrolysis conditions, and reactor configuration, making optimization essential for maximizing adsorption performance (Huang et al. 2020; Wang et al. 2024).

However, pristine biochars generally exhibit limited phosphate adsorption capacities due to the predominantly negative surface charge and low abundance of active metal sites. Consequently, numerous studies have focused on modifying biochar surfaces with metal species such as Mg, Ca, Fe, Al, Zn, and La to enhance phosphate affinity and adsorption performance (Panwar and Pawar 2020; Yadav et al. 2022).

Recent advances have demonstrated significant improvements in phosphate adsorption through the development of metal-modified biochars and multifunctional sorbents designed not only for phosphate removal but also for phosphorus recovery and reuse as slow-release fertilizers. In particular, calcium-modified biochars have attracted increasing attention because calcium species promote phosphate immobilization through surface precipitation and complexation mechanisms, resulting in high adsorption efficiencies and facilitating nutrient recycling. These developments align with broader efforts to transform low-value biomass residues into high-value functional materials capable of contributing to circular nutrient management (Nadagouda et al. 2024; Zhang et al. 2025; Zheng et al. 2023).

Recent studies have reported phosphate adsorption capacities exceeding 50–200 mg g⁻^1^ for various metal-modified biochars, particularly those enriched with Mg, Fe, La, and Ca species. Calcium-modified biochars have emerged as particularly attractive alternatives because they combine relatively low-cost modification procedures with strong phosphate immobilization through the formation of calcium phosphate phases, facilitating subsequent phosphorus recovery and reuse. Nevertheless, direct comparisons among different production scales and reactor configurations remain scarce, limiting the understanding of how manufacturing conditions affect adsorption performance and practical applicability (Abeysinghe et al. 2026; Deng and Dhar 2023; Feng et al. 2022; Liu et al. 2024; Yu et al. 2022).

Despite the growing body of literature on phosphate adsorption by modified biochars, three important gaps can still be identified. First, relatively few studies have focused on calcium-modified biochars derived from pine sawdust, despite the abundance and industrial relevance of this biomass. Second, most investigations remain restricted to laboratory-scale batch pyrolysis systems, while pilot-scale continuous-flow production has received considerably less attention. Third, the combined effects of modification parameters and production conditions are rarely evaluated through statistically robust experimental design approaches. These limitations hinder the transfer of promising laboratory results to scalable and economically viable phosphorus recovery technologies.

Therefore, this study investigated the production of calcium-modified pine sawdust biochars using both laboratory-scale tubular and pilot-scale continuous-flow pyrolysis systems. A 2^3^ factorial experimental design was applied to optimize impregnation time, CaCl_2_ concentration, and biomass mass, aiming to maximize phosphate adsorption performance. The resulting materials were comprehensively characterized and evaluated for phosphate removal from aqueous solutions. By simultaneously addressing feedstock selection, chemical modification, statistical optimization, and production scale-up, this work provides one of the few systematic assessments of calcium-modified biochar production from laboratory to pilot scale, contributing to the development of scalable technologies for phosphorus recovery and eutrophication mitigation.

## Materials and methods

### Biomass modification and pyrolysis

Pine sawdust, a by-product from the wood-processing industry in Paraná (Brazil), was selected as the raw biomass for biochar production. The material was first dried in the oven at 100 °C for 24 h (Gehaka, G3), and subsequently ground using a knife mill (Fortninox, STAR FT-50), with a 28 mesh, to obtain a homogeneous particle size suitable for chemical modification. The biomass was modified with 50 mL CaCl_2_ (Exodo Cientifica, > 99% purity) and the modification followed a 2^3^ factorial experimental design, with the following independent variables: time (*t* = 1 or 2 h), concentration of CaCl_2_ solution (*c* = 0.5 or 1.0 mol L⁻^1^), and biomass mass (*m* = 5 or 10 g). Eight experiments were carried out in total, ensuring evaluation of both main and interaction effects among the variables. The dependent variable was % phosphate removal. The factors and coded values are listed in Table 1.

**Table 1:** Factors and codes of 2^3^ experimental design matrix

Coded values	Factor A: time (h)	Factor B: concentration (mol L^−1^)	Factor C: biomass mass (g)
−1	1	0.5	5
+ 1	2	1.0	10

After impregnation, the samples were filtered under vacuum, and dried at 105 °C for 12 h (Gehaka, G3). The factorial design allowed systematic optimization of the modification process by quantifying the relative contributions of each parameter and their interactions on phosphate removal efficiency. The analysis of variance (ANOVA) tested the statistical significance of the independent variables and their interactions at *p*-value < 0.05 with a 95% confidence level. The parameters *F*-value, predicted R-squared (*R*^2^_pred_), adjusted R-squared (*R*^2^_adj_), and predicted residual error sum of squares were evaluated. The biochars were prepared according to the experimental design conditions in Table 2 in a tubular furnace (EDG, FT-HI/40) with heating rate of 5 °C min^−1^, temperature of 450 °C and no residence time (da Rocha et al. 2024).

**Table 2:** Percentage of phosphate removal under a 2^3^ experimental design

Samples	Variables			Percentage of removal (%)
	t (h)	c (mol L^−1^)	m (g)	
1	1	0.5	5	31 ± 1
2	2	0.5	5	28 ± 1
3	1	1.0	5	87 ± 2
4	2	1.0	5	93 ± 3
5	1	0.5	10	13 ± 2
6	2	0.5	10	87 ± 3
7	1	1.0	10	18 ± 3
8	2	1.0	10	97 ± 3

In the end of the pyrolysis process, the reactor was cooled to room temperature and the biochars were weighed. After the sorption experiments, two conditions (from the eight experiments) were chosen to be pyrolyzed in two different systems: the laboratory-scale tubular furnace and a pilot-scale continuous flow furnace. In the tubular furnace, the samples were prepared with a heating rate of 5 °C min⁻^1^, temperature of 450 °C with no residence time at maximum temperature. This configuration provided high control over process parameters. In the continuous flow furnace, designed for larger-scale applications, the samples were produced under the same maximum temperature (450 °C) with feeding flow of 1 rpm, N_2_ flow of 4 L min⁻^1^, rotation of 8 rpm, and an inclination of 6 cm. The nomenclature of the resulting biochars followed the scheme: BSCa1T, BSCa2T, BSCa1F, BSCa2F, where B = biochar, S = sawdust, Ca = calcium, 1 or 2 = modification time, and T/F = tubular or continuous flow furnace.

### Phosphate sorption

Phosphate adsorption tests were conducted with an initial solution of 10 mg L⁻^1^ KH_2_PO_4_ (Sigma-Aldrich, > 99%), representative of polluted aquatic environments. In each experiment, 0.1 g of each biochar was mixed with 50 mL of the solution and maintained under continuous stirring for 24 h at 25 °C in an orbital shaker table (New Lab, NL-228/01). After contact, suspensions were filtered through a 0.45 µm pore size membrane of cellulose acetate, and the residual phosphate concentration was determined with a UV spectrophotometer (Shimadzu 2401 PC) using the molybdenum blue method at 880 nm (Baird et al. 2017). The experiments were performed in triplicates and the average values were presented. The phosphate sorbed by the biochars (q) and the corresponding removal percentage were calculated by Eqs. 1 and 2.

1 $$q=(\frac{{C}_{o}- {C}_{f} }{m}) V$$

2 $$\% \,Removal= \frac{{C}_{o}- {C}_{f} }{{C}_{o}} 100$$

- *q* is the phosphate sorbed by the biochar in mg g^−1^.
- *C*_o_ is the initial concentration of the phosphate in contact with the biochar in mg L^−1^.
- *C*_f_ is the concentration of the phosphate in the solution after the biochar sorption.
- *V* is the initial volume of the solution in L, and *m* is the sorbent mass in g.

The effect of competing anions on phosphate adsorption was evaluated in the presence of Cl⁻, NO_3_⁻, and SO_4_^2^⁻. Adsorption experiments were conducted under the optimized conditions using phosphate solutions containing the respective competing anions. After 24 h of contact time, the residual phosphate concentration was determined and the adsorption capacity was calculated.

For equilibrium adsorption studies, phosphate concentrations varied from 1 to 2000 mg L⁻^1^. The data were fitted to Langmuir (Eq. 3), Freundlich (Eq. 4), and Sips (Eq. 5) models to assess adsorption capacity and surface heterogeneity.

The Langmuir adsorption equilibrium equation is given by:

3 $${q}_{e}=\frac{{q}_{max}{K}_{L}{C}_{e} }{1+ {K}_{L}{C}_{e}}$$

- *q*_e_ is the amount of solute adsorbed per unit mass of adsorbent at equilibrium (mg g^−1^).
- *q*_max_ refers to the maximum adsorption capacity of the material (mg g^−1^).
- *K*_L_ is the Langmuir equilibrium constant (L mg^‐1^).
- C_e_ is the sorbate concentration in solution at equilibrium (mg L^−1^).

The Freundlich adsorption equilibrium equation is given by:

4 $${q}_{e}= {K}_{F}{C}_{e}^\frac{1}{n}$$

- *q*_e_ is the amount of solute adsorbed per unit mass of adsorbent at equilibrium (mg g^−1^).
- *C*_e_ is the sorbate concentration in solution at equilibrium (mg L^−1^).
- *K*_F_ is the Freundlich constant or adsorption coefficient [mg g⁻^1^ (mg L⁻^1^)⁻^1^/n].
- *n* is the Freundlich exponent (dimensionless).

The Sips adsorption equilibrium equation is given by:

5 $${q}_{e}=\frac{{q}_{max}{K}_{S}{C}_{e}^{n} }{1+ {K}_{S}{C}_{e}^{n}}$$

- *q*_e_ is the amount of solute adsorbed per unit mass of adsorbent at equilibrium (mg g⁻^1^).
- *q*_max_ refers to the maximum adsorption capacity (mg g⁻^1^).
- *K*_S_ is the Sips constant (L mg⁻^1^).
- *n* is the exponent that describes the degree of non-linearity of the system (dimensionless).
- *C*_e_ is the sorbate concentration in solution at equilibrium (mg L⁻^1^).

### Characterization

The biochars were characterized by a Fourier Transform Infrared Spectrometer (FT-IR, Bruker Vertex 70) to identify surface functional groups before and after pyrolysis. Potassium bromide was used as reference material and the samples were analyzed in the range 4000–400 cm^−1^, with 4 cm^−1^ and 32 scans of resolution. Scanning Electron Microscopy (SEM) and Dispersive Energy Spectroscopy (EDS) verify the samples morphology, with a scanning electron microscope (TESCAN, model VEGA3 LMU), with 15 kV in high vacuum. The samples were placed on supports with copper surfaces, followed by application of a thin layer of gold. The pores characterization was also obtained using BET (Nova 2000e, QuantachromeCo), at liquid nitrogen temperature. Raman spectroscopy (Witec Alpha 300R, *λ* = 532 nm) was employed to evaluate the structural ordering of the carbonaceous matrix, with particular attention to the I_D_/I_G_ ratio (intensity of defect to graphite bands). These analyses elucidated potential mechanisms of phosphate adsorption. The X-ray diffraction (XRD) data were obtained in a Shimadzu diffractometer model XRD 7000 (using copper radiation), from 5 to 80° θ with a 2° min^−1^ step. The point of zero charge (pH_PZC_) was determined using the pH drift method. Briefly, 50 mL of 0.1 mol L⁻^1^ NaCl solutions were adjusted to initial pH values ranging from 2 to 12 using HCl and NaOH solutions. Subsequently, 0.1 g of biochar was added to each solution, and the suspensions were shaken for 24 h at 25 °C. The pH_PZC_ was obtained from the intersection between the initial and final pH values.

## Results and discussion

### Optimization by factorial design

The results of the 2^3^ factorial design are presented in Table 2, showing phosphate removal efficiencies ranging from 13 to 97%.

Among the eight experiments performed, four achieved removal rates equal to or higher than 87%. The highest value (97%) was obtained under the condition where all factors were set at their highest levels (time: 2 h, concentration: 1.0 mol L⁻^1^, and mass: 10 g). Next, the condition with 2 h of contact time, 1.0 mol L⁻^1^ concentration, and 5 g of mass achieved 93% removal. Two other combinations also showed 87% removal: one involving 1 h of contact time, 1.0 mol L⁻^1^ concentration, and 5 g of mass, and another with 2 h of contact time, 0.5 mol L⁻^1^ concentration, and 10 g of mass. These results demonstrate that, under certain conditions, the increase of one factor (such as concentration) can compensate for another kept at a lower level (such as time), highlighting the importance of interactions among variables.

The analysis of the Pareto chart allowed the identification of statistically significant effects on removal efficiency. At a 95% confidence level (*p* < 0.05), all main effects (time, concentration, and mass) and second-order interactions (time × concentration, time × mass, and concentration × mass) were significant. Only the third-order interaction (time × concentration × mass) was not statistically significant. Among the positive effects, time (+ 39.0), concentration (+ 34.1), and the interaction time × mass (+ 37.4) stood out, indicating that increasing these factors substantially enhances removal efficiency. In contrast, the main effect of mass (− 6.1) and the concentration × mass interaction (− 26.6) were negative, suggesting that, in certain combinations, increasing the mass can reduce efficiency and that high concentrations combined with greater mass are not favorable.

Based on the significant effects, a mathematical model (Eq. 6) was fitted, with the independent variables coded as *X*_1_ (time), *X*_2_ (concentration), and *X*_3_ (mass):

6 $$Removal\;\left(\%\right)=56.75+19.52X_1+17.07X_2-3.04X_3-18.69X_1X_3-13.28X_2X_3+1.63X_1X2$$

The model showed excellent goodness of fit, explaining 99.7% of the variability in the response (*R*^2^ = 0.9973; adjusted *R*^2^ = 0.9961) and high predictive power (predicted *R*^2^ = 0.9938). The standard error of regression was low (*S* = 2.23), indicating good precision. The regression was statistically significant (*p* < 0.01), confirming the robustness of the fit.

Among the independent variables analyzed, modification time was the most influential positive factor, followed by initial concentration, both showing strong and favorable effects on removal. The negative effect of mass, although significant, was offset by its positive interaction with time, suggesting that higher masses enhance removal only when combined with longer modification times. On the other hand, the concentration × mass interaction was negative, reinforcing that the efficiency gain from increasing concentration is attenuated when mass is also set at a high level. In general, the results obtained from analyses reinforce the importance of the interactions among the variables in the experimental design. The concentration of the modification solution was the most influential factor in phosphorus removal, but its association with time and mass was also decisive for the material’s performance. Thus, the statistical analysis combined with the experimental design not only contributed to understanding the effects but also guided the optimization of the parameters selected for biochar modification.

### Comparison of pyrolysis furnaces

Selected samples (1.0 mol L^−1^, 5 g of biomass and modification time of 1 and 2 h) were pyrolyzed in both tubular and continuous flow furnaces to assess scalability. The nomenclature of the samples was BSCa1T, BSCa2T, BSCa1F, BSCa2F, where B = biochar, S = sawdust, Ca = calcium, 1 or 2 = modification time, and T/F = tubular or continuous flow furnace.

Phosphate removal efficiencies were 87% (BSCa1T), 75% (BSCa1F), 93% (BSCa2T), and 91% (BSCa2F). The similarity between tubular and continuous-flow results demonstrates the feasibility of scaling up biochar production without compromising adsorption efficiency. This is a key outcome since industrial implementation requires large-scale continuous processes, and many studies remain restricted to laboratory-scale production.

### Biochar characterization

**Fig. 1:**
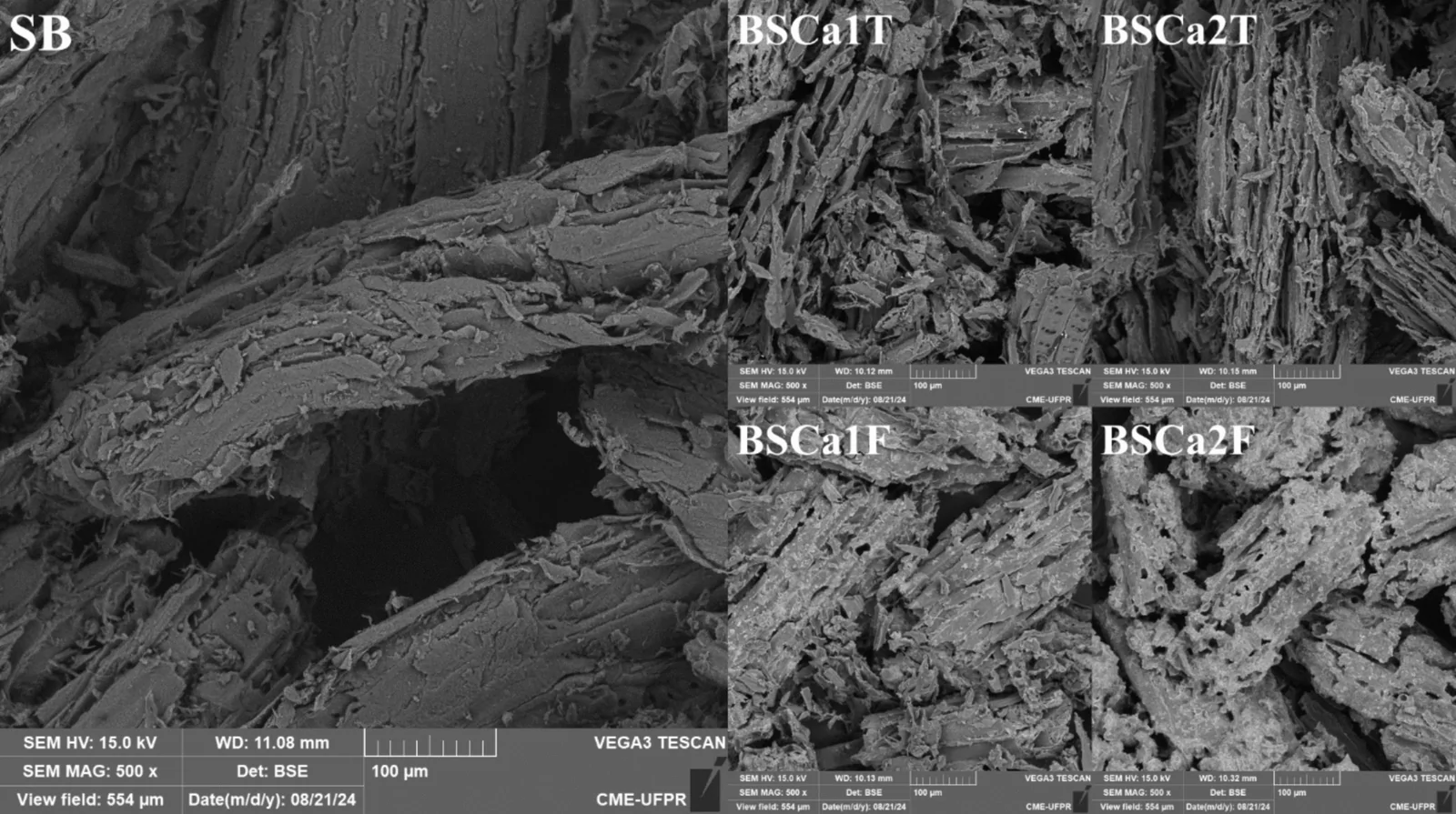
SEM images of BS, BSCa1T, BSCa2T, BSCa1F, and BSCa2F

The morphological features of the precursor biomass and derived biochars were examined by SEM to evaluate the structural transformations induced by pyrolysis (Fig. 1). The raw pine sawdust presented a fibrous morphology with compact and thick cell walls, typical of lignocellulosic materials such as wood. The surface displayed ordered cellular structures associated with the presence of lignin, cellulose, and hemicellulose, which contribute to the rigidity and integrity of the biomass matrix (Benyoucef et al. 2020; Mzinyane et al. 2023). Only a few natural fissures and pores were observed, reflecting the limited inherent porosity of the untreated biomass.

After pyrolysis, significant morphological changes became evident in the biochars. The micrographs revealed irregular, heterogeneous surfaces containing fissures, cracks, and collapsed structures, which are characteristic of thermally carbonized materials (Ali et al. 2022; Zhou et al. 2024). These alterations are attributed to the devolatilization of cellulose, hemicellulose, and lignin, where the release of volatiles creates channels and pores, while dehydration, dehydrogenation, and decarboxylation reactions promote the loss of hydrogen-, oxygen-, and nitrogen-containing functional groups, leaving behind a carbon-rich matrix (Maaoui et al. 2024; Rijo et al. 2024).

Comparing the two pyrolysis systems, distinct differences were observed. Biochars produced in the tubular furnace, under gradual and controlled heating, tended to preserve elements of the original fibrous structure, showing a more defined morphology with less extensive collapse. In contrast, biochars from the continuous flow furnace exhibited more pronounced structural disruptions and severe collapses, suggesting that faster heating rates and dynamic feed conditions intensify the degradation of the biomass structure (Maaoui et al. 2024; Sawargaonkar et al. 2024). Despite these differences, both systems generated porous materials, confirming the suitability of both furnaces for producing adsorbents.

Overall, the SEM images confirm that pyrolysis induces the formation of fissures, cracks, and porosity, which are advantageous for adsorption applications. The observed morphological differences between furnaces highlight the interplay between process conditions and feedstock characteristics in defining the surface structure of biochars.

EDS analysis (Table 3) complemented by providing the elemental composition of the materials. Carbon and oxygen were the dominant elements in the biomasses, consistent with their lignocellulosic nature, while the biochars showed a relative decrease in oxygen content, reflecting the loss of oxygenated groups during pyrolysis (Călin et al. 2024). In addition, calcium associated with the chemical modifications was detected. The spatial distribution of these elements across the surface further demonstrated the effectiveness of the pre-pyrolysis impregnation process. Homogeneous dispersion indicates good interaction between the modifying salts and the carbonaceous matrix, whereas localized clusters suggest partial migration or precipitation during heating. Together, the SEM and EDS results confirm that pyrolysis promotes profound morphological changes and enables the incorporation of modifying agents, both of which enhance the surface reactivity of biochars and their potential as phosphate adsorbents.

**Table 3:** Average percentage values of the elements detected in the images of sawdust biomass (BS) and biochars

Samples	Mean %					
	C	O	Cl	Ca	Al	Fe
BS	58	42	ND	ND	ND	ND
BSCa1T	65	19	8	8	ND	ND
BSCa1F	65	16	10	8.5	0.1	ND
BSCa2T	66	17	8	8	ND	ND
BSCa2F	52	17	17.5	12	0.2	0.6

*ND* non-detected

To further investigate the textural properties of the calcium-modified biochars, N_2_ adsorption-desorption analyses were performed. The pore characteristics are presented in Table 4.

**Table 4:** Pore characteristics of the biochars indicating the surface area (SA), the pore volume and the average diameter

Samples	SA (m^2^ g^−1^)	Pore volume (cm^3^ g^−1^)	Average diameter (nm)
BSCa1T*	-	-	-
BSCa1F	4.53	0.019	17.4
BSCa2T	6.95	0.025	14.4
BSCa2F	7.14	0.027	15.0

*The sample did not adjust due to the low value of SA

The BET surface areas obtained for the valid samples ranged from 4.53 to 7.14 m^2^ g⁻^1^, while the total pore volumes varied between 0.019 and 0.027 cm^3^ g⁻^1^. These values indicate that the materials possess relatively low specific surface areas, despite the presence of fissures, cracks, and porous regions observed by SEM. The average pore diameters ranged from 14.4 to 17.4 nm, characterizing the materials as predominantly mesoporous.

The increase in modification time from 1 to 2 h promoted a slight increase in both surface area and pore volume, particularly for BSCa2F, which presented the highest BET surface area and pore volume among the evaluated biochars. However, the overall differences between samples remained relatively small, suggesting that the pyrolysis system and modification time exerted only a limited influence on the development of extensive surface area.

The low BET surface areas observed are consistent with values commonly reported in the literature for calcium-modified biochars derived from sawdust (da Rocha et al. 2024). Yang et al. (2023) produced biochars by co-pyrolyzing sawdust and peanut shell with phosphate tailings to enhance heavy metal removal from water. The SAs of sawdust biochar ranged from 9.36 to 11.6 m^2^ g⁻^1^, while also enriching the material with phosphorus-containing mineral phases and functional groups.

The incorporation of calcium species may partially occupy pores and cover portions of the carbon surface, limiting the development of highly porous structures while simultaneously creating chemically active sites for phosphate sorption (Abeysinghe et al. 2026). Therefore, although the porous structure contributes to mass transfer and facilitates the accessibility of phosphate ions to reactive sites, the sorption process is not expected to be primarily governed by physisorption mechanisms associated with high surface area (Pattanaik et al. 2025).

This interpretation is further supported by the phosphate removal efficiencies observed for the biochars. If surface area were the dominant factor controlling sorption, substantially higher BET values would be expected. This suggests that chemical interactions involving calcium-containing phases play a predominant role in phosphate uptake (Pattanaik et al. 2025). Consequently, the textural properties of the biochars appear to act mainly as auxiliary features that improve accessibility to sorption sites, whereas calcium-mediated mechanisms are responsible for the major contribution to phosphate removal.

**Fig. 2:**
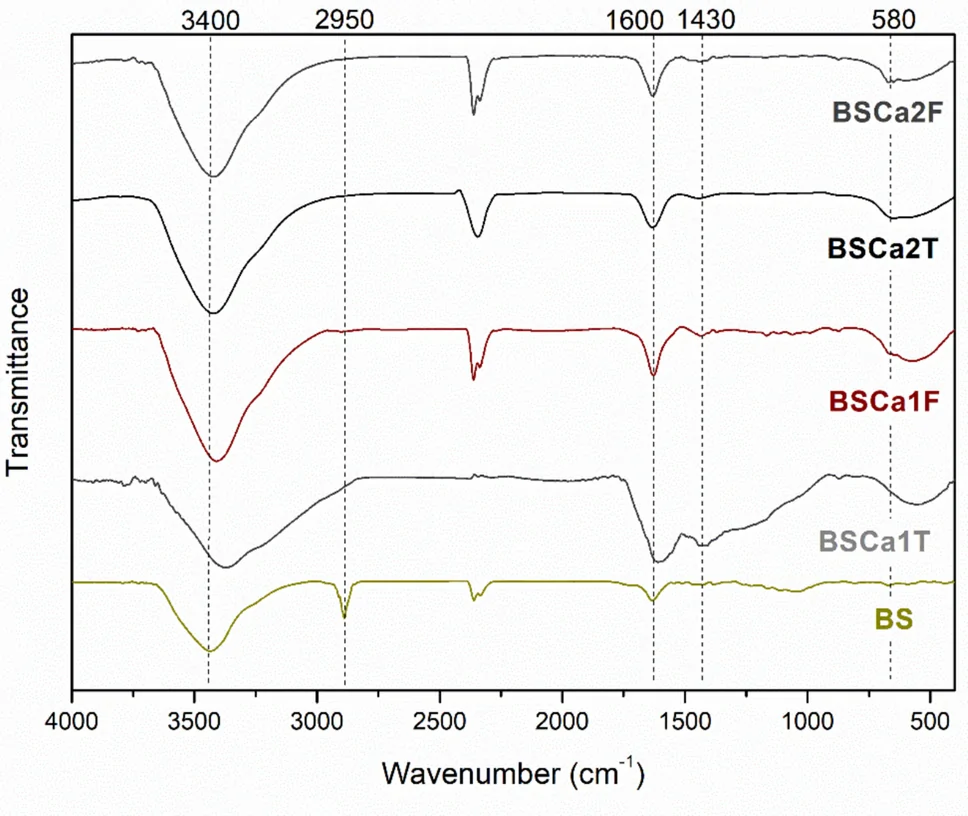
FTIR spectra of BS, BSCa1T, BSCa2T, BSCa1F, and BSCa2F

The FTIR spectra of sawdust and derived biochars (Fig. 2) revealed a general similarity in functional groups, even between materials produced in different furnaces, indicating that pyrolysis did not eliminate surface functionalities. The broad band observed at 3350–3700 cm⁻^1^ corresponds to O–H stretching vibrations, associated with hydroxyl groups from glucose units in cellulose and hemicellulose, as well as phenolic functionalities from lignin, and is often related to hydrogen bonding (Chen et al. 2024; Yang et al. 2025). These hydroxyl groups are important because they provide active sites capable of interacting with phosphate species through electrostatic attraction and ligand exchange. In the sawdust spectrum, the region at 2900–2990 cm⁻^1^ shows symmetric and asymmetric C–H stretching vibrations of aliphatic groups (CH₂ and CH₃). These bands disappeared almost entirely in the biochar spectra, reflecting the decomposition of aliphatic C–H bonds during pyrolysis (Qing et al. 2024).

The bands located at 1650–1750 cm⁻^1^ are attributed to C = O stretching vibrations, which can be associated with carboxylic acids, esters, ketones, and aldehydes, and indicate partial oxidation of the organic matrix. Adjacent regions between 1400 and 1650 cm⁻^1^ can also correspond to C = C stretching vibrations of unsaturated and aromatic structures (Rohman et al. 2024). The band at 1300–1000 cm⁻^1^, present in sawdust, is assigned to C–O–C stretching of polysaccharides such as cellulose, hemicellulose, and lignin. This band is significantly less intense in biochars, reflecting the reduction of the lignocellulosic fraction after pyrolysis (Yang et al. 2025). Overall, the persistence of oxygen-containing groups (–OH, C = O, C–O–C) in the biochars, regardless of the furnace used, is of particular interest, since these groups play a fundamental role in phosphate adsorption mechanisms. Their presence suggests that the materials retain reactive sites capable of interacting with phosphate ions, supporting the suitability of calcium-modified biochars as efficient adsorbents.

**Fig. 3:**
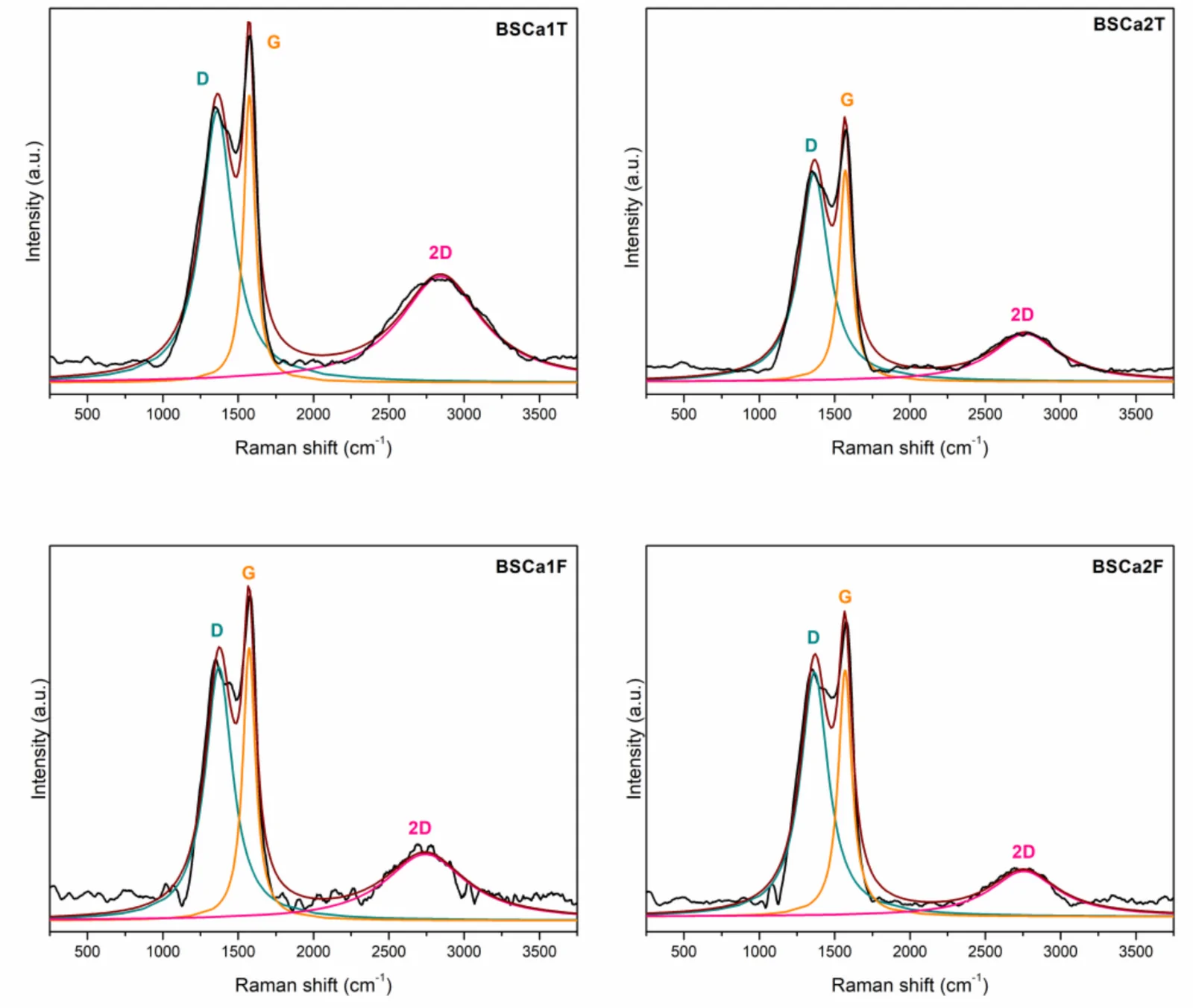
Raman spectra of BSCa1T, BSCa2T, BSCa1F, and BSCa2F

Raman spectroscopy was employed to investigate the structural organization of the carbonaceous matrix in the biochars, providing insights into the degree of order and the presence of defects (Masuku et al. 2024). All samples (Fig. 3) exhibited the characteristic D, G, and 2D bands, typically observed in carbon-based materials.

The D band, located at 1300–1400 cm⁻^1^, is associated with defects and disorder in the carbon structure, which may originate from sp^2^ bond breakage, structural flaws, and the incorporation of heteroatoms or impurities (Deng et al. 2024). The G band, at 1500–1600 cm⁻^1^, corresponds to the E_2g_ vibrational modes of sp^2^-hybridized carbon atoms in aromatic rings, and its presence indicates the development of graphitic domains (Makowska and Dziosa 2024). The 2D band, observed between 2500 and 3000 cm⁻^1^, is a second-order feature of the D band and is often linked to the formation of graphitic layers and graphene-like structures (Veiga et al. 2021). The appearance of these three bands in the biochars, absent in the raw sawdust due to fluorescence interference, confirms the effectiveness of the pyrolysis process in transforming amorphous biomass into partially graphitized carbon.

The structural defects were evaluated using the ratio of the D to G band areas (I_D_/I_G_), a parameter for assessing the degree of order in carbonaceous materials. Lower I_D_/I_G_ values indicate fewer defects and greater similarity to the graphite structure, whereas higher ratios suggest a more disordered, amorphous character (Thairattananon et al. 2024). The I_D_/I_G_ ratios obtained were 2.62 (BSCa1T), 2.35 (BSCa2T), 2.01 (BSCa1F), and 2.01 (BSCa2F). These results show that, despite the more controlled conditions of the tubular furnace, biochars produced in the continuous flow furnace exhibited comparable or even lower structural disorder, reflecting a higher degree of organization. This finding is significant because it demonstrates that pilot-scale production is not only feasible but can yield materials with structural quality on par with laboratory-scale processes.

Overall, the presence of defects indicated by the D band, combined with the persistence of graphitic domains represented by the G and 2D bands, highlights the dual nature of biochars as partially ordered but defect-rich materials. Such structural features are advantageous for adsorption applications, as defects and oxygen-containing sites can act as active centers for phosphate binding, while the graphitic domains ensure stability of the material during environmental applications.

**Fig. 4:**
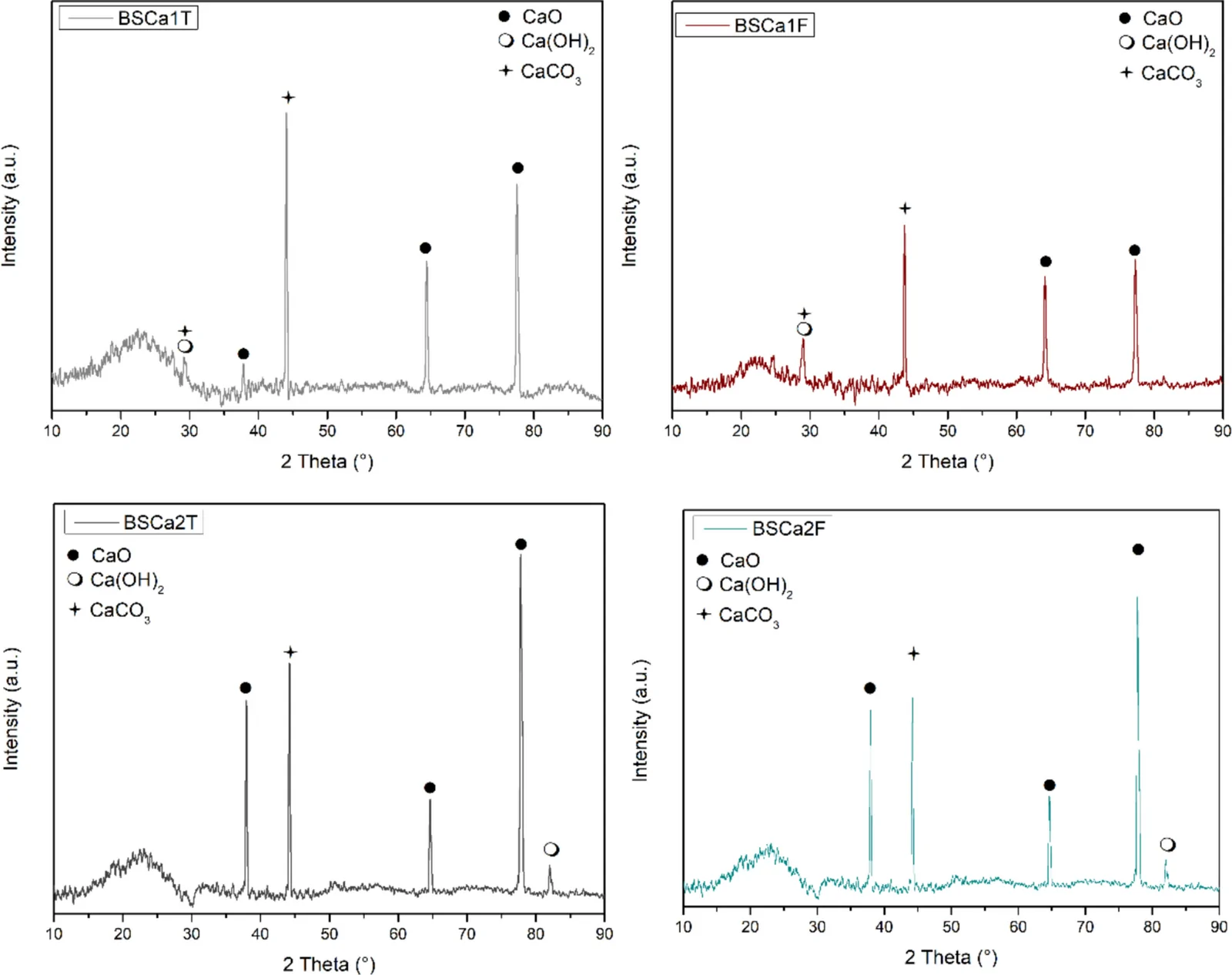
Diffractograms of BSCa1T, BSCa1F, BSCa2T, and BSCa2F

X-ray diffraction analysis was performed to identify the crystalline phases present in the calcium-modified biochars and to provide evidence regarding the potential mechanisms involved in phosphate sorption. The diffractograms of BSCa1T, BSCa2T, BSCa1F, and BSCa2F (Fig. 4) revealed the presence of sharp diffraction peaks superimposed on a broad halo between 2θ = 20–30°, characteristic of the amorphous carbonaceous matrix typically observed in biochars (Wang et al. 2021).

The crystalline phases identified were mainly associated with calcium species, including CaO, Ca(OH)_2_, and CaCO_3_. Diffraction peaks located at approximately 2θ = 36°, 38°, 64°, and 78° were attributed to CaO (ICSD: 148208), while peaks near 29°, 36°, and 82° indicated the presence of Ca(OH)_2_ (ICSD: 51411). Additional reflections around 25°, 29°, and 44° confirmed the occurrence of CaCO_3_ (ICSD: 137396). The formation of these phases is expected during pyrolysis due to the thermal transformation of the calcium precursor and subsequent reactions with atmospheric CO_2_ (Lei et al. 2017; Wang et al. 2021).

The identification of these calcium-bearing phases provides direct evidence for the active sites responsible for phosphate removal. Calcium oxides and hydroxides can promote phosphate sorption through surface complexation reactions involving hydroxyl groups, while dissolved Ca^2^⁺ released from these phases may induce the precipitation of poorly soluble calcium phosphates (Lei et al. 2017; Wang et al. 2021; Xu et al. 2022). Therefore, the occurrence of CaO, Ca(OH)_2_, and CaCO_3_ strongly supports the predominance of precipitation and surface complexation mechanisms during phosphate uptake.

These findings complement the FTIR and EDS results, which confirmed the presence of oxygen-containing functional groups and calcium distributed throughout the biochar surface. However, the identification of these phases alone does not fully explain the sorption behavior. Besides the availability of calcium-containing active sites, the surface charge of the biochars also plays an important role in controlling the interaction between phosphate species and the adsorbent surface. Therefore, the determination of the pH_PZC_ provides complementary information regarding the electrostatic properties of the materials and helps to clarify the contribution of surface charge effects to phosphate sorption.

**Fig. 5:**
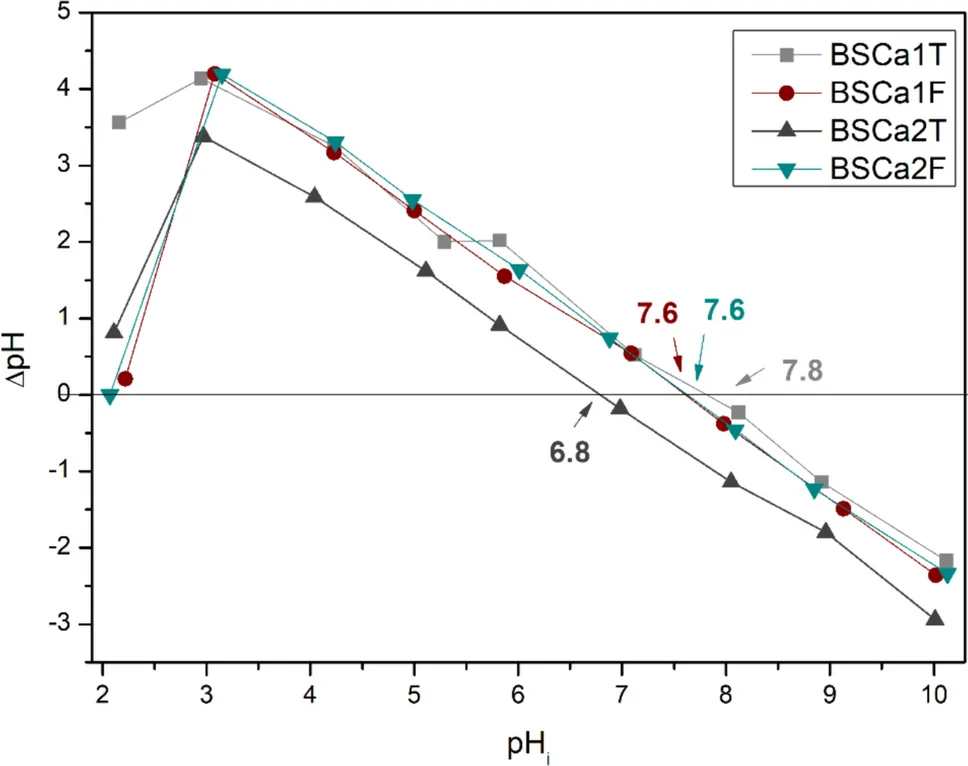
pH_PZC_ for BSCa1T, BSCa1F, BSCa2T, and BSCa2F

The pH_PZC_ values obtained for BSCa1T, BSCa1F, BSCa2T, and BSCa2F (Fig. 5) were 7.8, 7.6, 6.8, and 7.6, respectively. The pH_PZC_ corresponds to the pH at which the net surface charge of the biochar is zero. At pH values below the pH_PZC_, the biochar surface becomes predominantly positively charged, favoring the sorption of anionic species. Conversely, at pH values above the pH_PZC_, the surface progressively acquires a negative charge, which may reduce the sorption of negatively charged species due to electrostatic repulsion (Abeysinghe et al. 2022; Bakatula et al. 2018).

The influence of pH on phosphate sorption is closely related to both surface charge and phosphate speciation. At pH ≤ 2, phosphate is predominantly present as neutral H_3_PO_4_, which exhibits limited interaction with the biochar surface. Consequently, the lowest phosphate removal efficiencies were observed under strongly acidic conditions. Between pH 2 and 7, phosphate exists mainly as H₂PO₄⁻, while the biochar surfaces remain positively charged due to the pH_PZC_ values being above the solution pH. Under these conditions, electrostatic attraction favors the approach of phosphate ions to the adsorbent surface, contributing to the removal efficiencies observed for most biochars (Deng and Dhar 2023; Liu et al. 2024).

As the pH increases above 7, HPO_4_^2^⁻ becomes the predominant phosphate species and the surface charge gradually shifts toward negative values as the solution pH approaches or exceeds the pH_PZC_. In principle, this condition should reduce phosphate sorption due to electrostatic repulsion. However, phosphate removal remained relatively high even under alkaline conditions. This behavior indicates that electrostatic attraction alone cannot explain the sorption process. Instead, the results suggest that calcium-mediated mechanisms, including surface complexation and calcium-phosphate precipitation, play a major role in phosphate uptake. The persistence of removal efficiencies across a broad pH range demonstrates that the calcium-modified biochars maintain effective sorption performance even when electrostatic conditions become less favorable (Deng and Dhar 2023; Liu et al. 2024).

The combined analysis of pH_PZC_ and pH-dependent sorption behavior indicates that electrostatic attraction contributes to phosphate removal, particularly under acidic and near-neutral conditions. Nevertheless, the sustained sorption observed at pH values above the pH_PZC_ highlights the importance of calcium-containing active phases, reinforcing the hypothesis that phosphate sorption occurs predominantly through surface complexation and calcium-phosphate precipitation mechanisms. Although electrostatic attraction alone cannot explain the sorption capacities observed, the pH_PZC_ results demonstrate that surface charge effects act synergistically with calcium-mediated mechanisms, increasing the accessibility of phosphate ions to hydroxylated calcium species identified by XRD and FTIR. Overall, the characterization results provide strong evidence regarding the mechanisms responsible for phosphate sorption onto the calcium-modified biochars.

### Proposed phosphate sorption mechanism

The phosphate sorption behavior observed for BSCa1T, BSCa1F, BSCa2T, and BSCa2F indicates that phosphate uptake is controlled by multiple mechanisms acting simultaneously. The higher removal efficiencies obtained for the biochars modified for 2 h, particularly BSCa2T (93%) and BSCa2F (91%), suggest that the longer modification period promoted the formation of a greater number of reactive calcium sites available for phosphate interaction.

Based on the characterization results, calcium-mediated mechanisms are expected to dominate the sorption process. The occurrence of calcium-containing phases, together with the preservation of oxygenated surface functionalities, creates favorable conditions for phosphate immobilization through surface complexation and precipitation reactions. In this process, phosphate species can interact directly with hydroxylated calcium sites located on the biochar surface, forming stable inner-sphere complexes. Simultaneously, calcium species associated with the carbon matrix may participate in the formation of insoluble calcium-phosphate compounds (Lei et al. 2017; Pattanaik et al. 2025; Wang et al. 2021; Xu et al. 2022).

Electrostatic attraction may also contribute to phosphate uptake. Since the pH_PZC_ values were close to neutral pH, the biochar surface tends to exhibit a positive charge under acidic and slightly neutral conditions, facilitating the initial approach of negatively charged phosphate species. However, considering the sorption capacities observed and the abundance of calcium phases identified in the materials, electrostatic attraction is likely a secondary mechanism that enhances phosphate transport toward the active sites rather than acting as the main removal pathway (Deng and Dhar 2023).

The structural characteristics of the biochars may further contribute to sorption performance. The presence of surface defects, porous regions, and a stable carbonaceous matrix increases the accessibility of phosphate ions to reactive calcium sites. These features become particularly important because they facilitate mass transfer and promote the interaction between dissolved phosphate species and the mineral phases dispersed throughout the biochar structure (Chen et al. 2022).

Therefore, the sorption mechanism proposed for the calcium-modified biochars involves an initial electrostatic attraction between phosphate species and the positively charged surface, followed by stronger chemical interactions through surface complexation and calcium-phosphate precipitation. Among these mechanisms, precipitation and complexation are expected to be the predominant pathways responsible for phosphate removal, as seen in the Eqs. 7, 8, and 9 (Lei et al. 2017; Pattanaik et al. 2025; Wang et al. 2021; Xu et al. 2022).

7 $$Ca-OH+H_2PO_4\rightarrow Ca-OPO_3+H_2O$$

8 $$3Ca^{2+}+6PO_{4^{3-}}\rightarrow Ca_3{\left(PO_4\right)}_2\left(s\right)$$

9 $$10Ca^{2+}+6PO_{4^{3-}}+2OH^-+2OH^-\rightarrow Ca_{10}{\left(PO_4\right)}_6\left(OH_2\right)\left(s\right)$$

Overall, the integration of FTIR, SEM/EDS, XRD, pH_PZC_, and Raman results provides consistent evidence for the proposed sorption mechanism. The presence of calcium-bearing mineral phases, oxygen-containing functional groups, favorable surface charge conditions, and structurally stable carbon matrices collectively supports phosphate immobilization through a combination of electrostatic attraction, surface complexation, and calcium-phosphate precipitation. Among these pathways, the formation of calcium-phosphate species and the interaction of phosphate with hydroxylated calcium sites are expected to be the dominant mechanisms responsible for the sorption capacities observed for the calcium-modified biochars.

### Competing anions

**Fig. 6:**
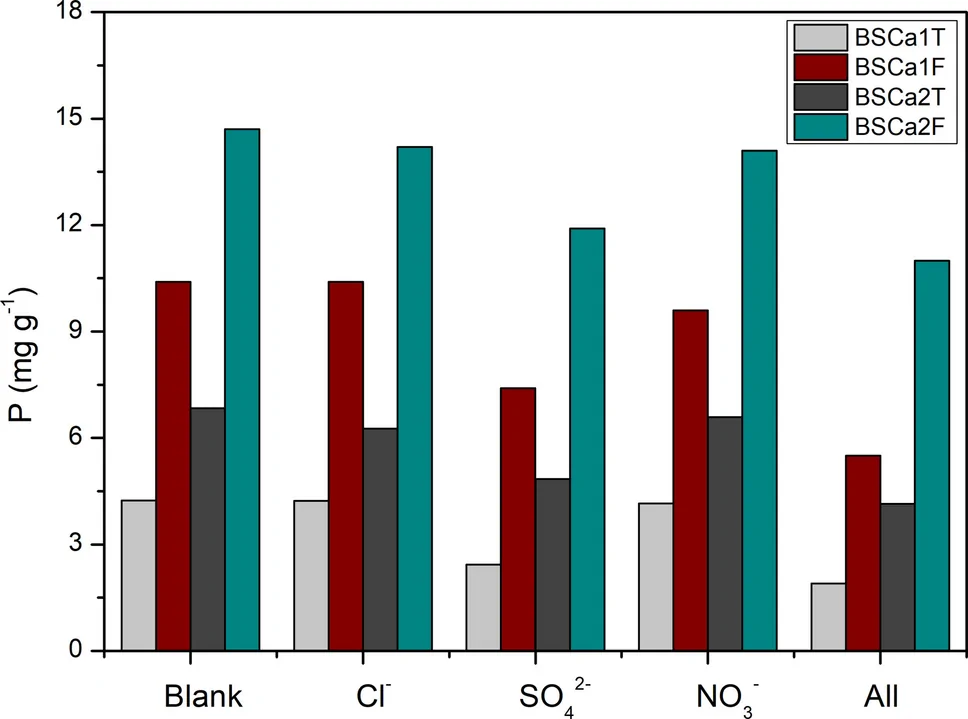
Effects of Cl⁻, SO_4_^2^⁻, and NO_3_⁻ on P sorption for BSCa1T, BSCa2T, BSCa1F, and BSCa2F

The effects of coexisting anions on phosphate sorption are presented in Fig. 6.

Since phosphate is commonly found in natural waters and wastewaters containing various dissolved ions, the influence of Cl⁻, SO_4_^2^⁻, and NO_3_⁻ was investigated to evaluate the selectivity of the produced biochars. Overall, the presence of competing anions affected phosphate sorption to different extents depending on the anion type and the biochar evaluated. However, all materials maintained considerable phosphate uptake capacities, indicating a strong affinity between phosphate species and the active sites generated by calcium modification (Yu et al. 2022).

For the tubular reactor biochars (BSCa1T and BSCa2T), sulfate caused the most pronounced decrease in adsorption capacity, reducing phosphate uptake from approximately 4.2 and 6.8 mg g⁻^1^ to 2.4 and 4.8 mg g⁻^1^, respectively. In contrast, chloride and nitrate produced only minor changes in adsorption performance. The behavior that SO_4_^2−^ had on phosphate sorption can be explained taking their ionic radius into account, where for H_2_PO_4_^−^ is 0.238 nm and SO_4_^2−^ is 0.230 nm (Missau et al. 2022). A similar trend was observed for the continuous-flow biochars (BSCa1F and BSCa2F), although the reduction was less pronounced. Even in the presence of competing ions, these materials maintained their adsorption capacities, particularly BSCa2F.

The greater interference caused by SO_4_^2^⁻ can be attributed to its divalent nature and its stronger interaction with calcium-containing active sites. Previous characterization results demonstrated the presence of CaO, Ca(OH)_2_, and CaCO_3_ phases on the biochar surface, suggesting that phosphate removal occurs predominantly through ligand exchange, surface complexation, and calcium phosphate precipitation. Sulfate ions may partially compete with phosphate for reactive calcium species or surface hydroxyl groups, reducing the availability of adsorption sites (Liu et al. 2023). In contrast, Cl⁻ and NO_3_^−^ are monovalent ions with lower affinity toward calcium-rich surfaces and therefore exerted only a limited competitive effect (Feng et al. 2022).

When all competing anions were simultaneously present, the adsorption capacity decreased for all biochars, indicating a cumulative competitive effect. Nevertheless, the reduction was moderate, especially for the continuous-flow materials. BSCa2F retained approximately 11 mg g⁻^1^ of phosphate adsorption capacity, corresponding to nearly 75% of the value obtained in the absence of competing ions. This behavior demonstrates the selectivity of the calcium-modified biochars toward phosphate, even under more complex aqueous conditions that better resemble real environmental matrices (Feng et al. 2022).

The relatively low influence of Cl⁻ and NO_3_^−^, together with the moderate impact of SO_4_^2^⁻, suggests that phosphate adsorption is not governed solely by electrostatic attraction. If electrostatic interactions were the dominant mechanism, a more significant reduction in adsorption would be expected in the presence of competing anions. Instead, the results reinforce the predominance of specific chemical interactions, including ligand exchange, surface complexation, and calcium phosphate precipitation, which are consistent with the findings obtained from XRD, FTIR, and pH_PZC_ analyses. These mechanisms provide the materials with phosphate selectivity and contribute to their promising performance for phosphate recovery from complex wastewater systems.

### Equilibrium study

**Fig. 7:**
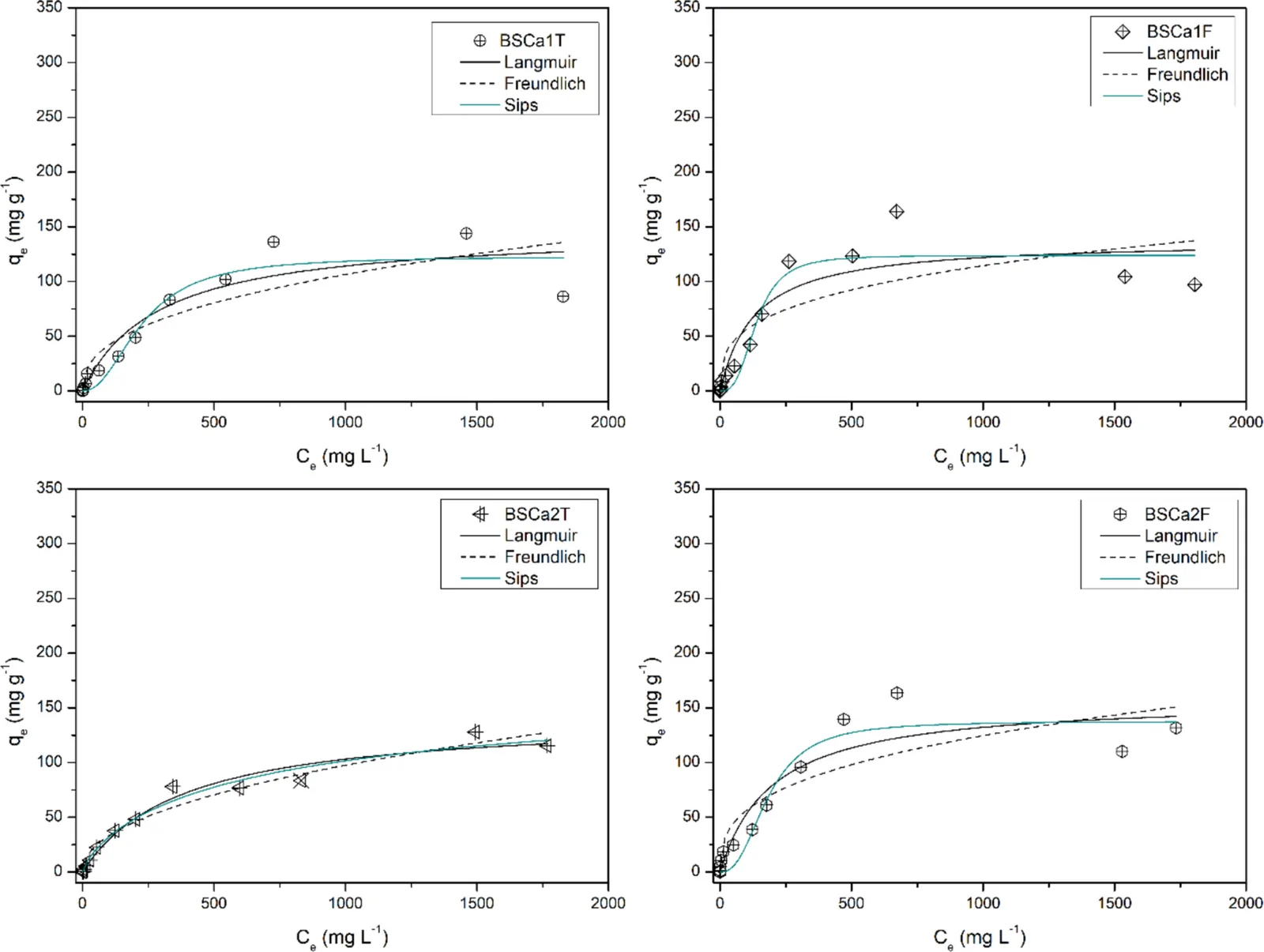
Equilibrium models for BSCa1T, BSCa1F, BSCa2T, and BSCa2F

The sorption capacity of the biochars was evaluated through equilibrium studies, which describe how phosphate molecules distribute between the liquid and solid phases once the system reaches equilibrium. Table 5 and Fig. 7 summarize the equilibrium parameters obtained for the Langmuir, Freundlich, and Sips models for the samples BSCa1T, BSCa1F, BSCa2T, and BSCa2F.

**Table 5:** Equilibrium parameters for Langmuir, Freundlich, and Sips

Biochars	Langmuir			Freundlich		Sips	
	***q***_**max**_** (mg g**^**−1**^**)**	***R***^**2**^	***R***_**L**_	**1/n**	***R***^**2**^	***n***	***R***^**2**^
BSCa1T	147.3	0.89	0.127	0.40	0.81	2.19	0.91
BSCa1F	137.9	0.85	0.062	0.31	0.71	3.00	0.90
BSCa2T	142.4	0.97	0.160	0.47	0.97	0.78	0.98
BSCa2F	158.4	0.90	0.090	0.35	0.81	2.50	0.92

The Langmuir model, which assumes monolayer adsorption on homogeneous surfaces, showed satisfactory fits for the evaluated biochars, with *R*^2^ values ranging from 0.85 to 0.97. Among the samples, BSCa2T presented the best adjustment to this model (*R*^2^ = 0.97), followed by BSCa2F (*R*^2^ = 0.90), BSCa1T (*R*^2^ = 0.89), and BSCa1F (*R*^2^ = 0.85) (Hameed et al. 2007; Manawi et al. 2024; Rwiza et al. 2018). The maximum adsorption capacities (*q*_max_) varied from 137.9 mg g⁻^1^ for BSCa1F to 158.4 mg g⁻^1^ for BSCa2F. The dimensionless separation factor (*R*_L_) ranged from 0.21 to 0.43, remaining within the interval 0 < *R*_L_ < 1, which confirms favorable phosphate adsorption onto all biochars. The relatively low *R*_L_ values suggest a strong affinity between phosphate ions and the adsorption sites generated by calcium modification.

The Freundlich model, which considers sorption on a heterogeneous surface also provided satisfactory fits, especially for BSCa2T (*R*^2^ = 0.97). The 1/n values ranged from 0.31 to 0.47, remaining below 1 for all samples, which indicates favorable adsorption and reflects the presence of heterogeneous adsorption sites with different interaction energies contributing to phosphate uptake. Such behavior suggests that, although calcium-rich phases play a major role in phosphate retention, surface heterogeneity also influences the sorption process (Hameed et al. 2007; Xia et al. 2016).

To better capture the complexity of the system, the Sips model provided the best overall description of phosphate adsorption, with *R*^2^ values ranging from 0.90 to 0.98. The highest fit was observed for BSCa2T (*R*^2^ = 0.98), followed by BSCa2F (*R*^2^ = 0.92), BSCa1T (*R*^2^ = 0.91), and BSCa1F (*R*^2^ = 0.90). These results indicate that phosphate adsorption onto the Ca-modified biochars is better represented by a hybrid model that combines characteristics of both homogeneous and heterogeneous adsorption. The Sips parameter n varied from 0.78 to 3.00, suggesting different degrees of surface heterogeneity among the biochars and reinforcing the coexistence of multiple adsorption environments (Saadi et al. 2015).

Overall, the results demonstrate that phosphate adsorption onto the evaluated biochars cannot be completely described by a single model. While the Langmuir model highlighted the high adsorption capacities and favorable adsorption behavior, the superior performance of the Sips model indicates that the sorption process occurs on surfaces containing both homogeneous and heterogeneous domains. This behavior is consistent with the presence of calcium-containing phases identified by physicochemical characterization, which promote phosphate removal through a combination of surface complexation and precipitation mechanisms.

The maximum phosphate adsorption capacities obtained in this study (137.9–158.4 mg g⁻^1^) are comparable to or higher than those reported for many metal-modified biochars available in the literature (Table 6).

**Table 6:** Comparison of phosphate sorption capacities reported for biochar-based adsorbents

Biochars	Feedstock/modification	*q*_max_ (mg g⁻^1^)	References
BSCa1T	Pine sawdust/Ca	147.3	This study
BSCa1F	Pine sawdust/Ca	137.9	This study
BSCa2T	Pine sawdust/Ca	142.4	This study
BSCa2F	Pine sawdust/Ca	158.4	This study
Ca-modified biochar	Peanut shell/Oyster	168.2	(Xu et al. 2022)
Ca-Al-La-modified biochar	Sawdust/Ca-Al-La	152.9	(Cheng et al. 2023)
Iron/calcium oxide-modified biochar	Bamboo/Fe-Ca	69.1	(Zeng et al. 2024)

The maximum phosphate adsorption capacities obtained in this study (137.9–158.4 mg g⁻^1^) demonstrate that the Ca-modified pine sawdust biochars are highly competitive compared with other engineered biochars reported in the literature (Table 6). Among the evaluated materials, BSCa2F exhibited the highest adsorption capacity (158.4 mg g⁻^1^), followed by BSCa1T (147.3 mg g⁻^1^), BSCa2T (142.4 mg g⁻^1^), and BSCa1F (137.9 mg g⁻^1^). These values are comparable to the adsorption capacity reported for a Ca-modified biochar derived from peanut shell and oyster shell (168.2 mg g⁻^1^), and for a Ca-Al-La modified sawdust biochar (152.9 mg g⁻^1^) (Cheng et al. 2023; Xu et al. 2022).

Notably, the adsorption capacities achieved in this study were substantially higher than those reported for Fe/Ca oxide-modified bamboo biochar (69.1 mg g⁻^1^) (Zeng et al. 2024), indicating that calcium modification of pine sawdust provides a highly effective strategy for phosphate removal. The superior performance may be associated with the formation of reactive calcium-containing phases, such as CaO, Ca(OH)_2_, and CaCO_3_, identified by XRD analysis, which promote phosphate retention through surface complexation and calcium-phosphate precipitation mechanisms.

Furthermore, while the Ca-Al-La modified biochar reported by (Cheng et al. 2023) required the incorporation of multiple metallic elements, including lanthanum, the biochars developed in the present study achieved similar adsorption capacities using only calcium as the modifying agent. This finding highlights the potential of the proposed material as a cost-effective and environmentally attractive alternative for phosphate recovery. Considering that the biochars were produced from an abundant lignocellulosic residue and pyrolyzed at a relatively low temperature (450 °C), the obtained adsorption capacities demonstrate the effectiveness of the modification strategy and reinforce the potential application of these materials within circular economy approaches for phosphorus recovery and water remediation.

The adsorption capacities obtained for the Ca-modified pine sawdust biochars place these materials among the most efficient Ca-based biochars reported in the literature, while maintaining a simpler synthesis route and employing low-cost biomass residues as feedstock.

## Conclusion

This study demonstrated that pine sawdust, an abundant industrial residue in southern Brazil, can be successfully transformed into calcium-modified biochars with high phosphate adsorption performance. The factorial design identified modification time, CaCl_2_ concentration, and biomass mass as the most influential variables affecting phosphate removal, while the interaction effects among these parameters played a critical role in optimizing adsorption efficiency. Under the optimized conditions, phosphate removal efficiencies reached up to 97%, confirming the effectiveness of the calcium modification strategy.

The comparison between tubular and continuous-flow pyrolysis systems showed that biochars produced in the pilot-scale continuous furnace achieved phosphate removal efficiencies comparable to those obtained in the laboratory-scale tubular reactor. These results demonstrate the feasibility of producing calcium-modified biochars under continuous operating conditions while maintaining adsorption performance, representing an important step toward future scale-up studies.

Characterization analyses revealed that pyrolysis and calcium impregnation promoted significant physicochemical modifications in the biomass. SEM, BET and EDS confirmed the development of fissures, cracks, and porous structures together with the successful incorporation of calcium species. FTIR indicated the persistence of oxygen-containing functional groups, Raman spectroscopy revealed partially ordered carbon structures with defect sites, and XRD confirmed the presence of CaO, Ca(OH)_2_, and CaCO_3_ phases. Combined with the pH_PZC_ and competing-anion results, these findings indicate that phosphate removal is governed predominantly by calcium-mediated mechanisms, particularly surface complexation and calcium-phosphate precipitation, while electrostatic interactions play a secondary role.

Equilibrium studies demonstrated favorable phosphate adsorption behavior, with the superior performance of the Sips model that indicates the sorption process occurs on surfaces containing both homogeneous and heterogeneous domains. The high adsorption capacities obtained highlight the strong affinity between phosphate species and the calcium-enriched surfaces generated during biochar production.

Overall, the results demonstrate that calcium-modified pine sawdust biochars are promising materials for phosphate removal and recovery from aqueous systems. Furthermore, considering that phosphate uptake occurs predominantly through the formation of calcium-phosphate species, the phosphorus-enriched biochar generated after adsorption may present potential for beneficial reuse as a phosphorus source or slow-release fertilizer, contributing to nutrient recycling and circular economy strategies.

## Data Availability

All data are included in the article. Additional information can be acquired with the corresponding author.
